# Planning for Retirement: Longitudinal Effect on Retirement Resources and Post-retirement Well-being

**DOI:** 10.3389/fpsyg.2017.01300

**Published:** 2017-07-27

**Authors:** Dannii Y. Yeung, Xiaoyu Zhou

**Affiliations:** Department of Applied Social Sciences, City University of Hong Kong Kowloon Tong, Hong Kong

**Keywords:** pre-retirement planning, retirement resources, psychological well-being, physical well-being, Chinese retirees

## Abstract

Retirement is a major life event, and a positive adjustment to retirement is essential for maintaining physical and psychological well-being in later life. Previous research demonstrates that pre-retirement planning predicts post-retirement well-being. This study further explores the underlying mechanism between planning activities and post-retirement well-being. By applying the resource-based dynamic model (Wang et al., 2011), the present longitudinal study examines whether pre-retirement planning activities can increase the total resources of retirees, including tangible, mental and social resources, and consequently contribute to better psychological and physical well-being 1 year after actual retirement. A total of 118 Hong Kong Chinese retirees completed three assessments: Time 1 assessment was conducted 6 months before retirement, and Times 2 and 3 assessments were carried out 6 and 12 months, respectively, after retirement. Latent growth models were employed to examine changes in retirement resources and post-retirement well-being over time. Consistent with the proposition of the resource-based dynamic model, positive changes in well-being were observed in the retirees with increases in retirement resources between pre- and post-retirement phases. The results of the latent growth mediation models also support our prediction: retirees with more preparatory activities before retirement acquire greater resources at the initial stage, which contribute to positive changes in post-retirement well-being over time.

## Introduction

Similar to other developed countries, the labor force of Hong Kong is rapidly aging. Over 70,000 civil servants are estimated to be retiring from their jobs in the coming decade (Hong Kong Government News, 2012). Retirement is a major life event, and a positive adjustment is essential for maintaining physical and psychological well-being in later life (Wang, 2007; Wong and Earl, 2009). The Health and Retirement Survey conducted in the United States revealed that roughly one in four retirees experiences a decline in psychological well-being 1-year after retirement (Wang, 2007; Wang and Bodner, 2007). However, majority of them do not report significant psychological changes over an 8-year period, and approximately 5% of them even showed improvement in psychological well-being. Identifying the protective factors of positive adjustment to this critical life transition is becoming increasingly important. Previous research demonstrates that pre-retirement planning is associated with post-retirement well-being (Reitzes and Mutran, 2004; Noone et al., 2013; Yeung, 2013), but the underlying mechanism between pre-retirement planning and successful adjustment remains largely unclear (Shultz and Wang, 2011). Therefore, the present study aims to fill this gap. Using the resource-based dynamic model (Wang et al., 2011) as the theoretical basis, retirees who perform more preparatory activities before retirement are hypothesized to possess greater amounts of resources, which contribute to better psychological and physical well-being after retirement.

### Resource-Based Dynamic Model

Hobfoll (2002) defined resources as the total capability of an individual to accomplish his/her valued needs and goals. Inspired by Hobfoll’s conservation of resources theory, retirement resources are broadly defined as various types of resources that are essential for retirement adjustment (Wang, 2007; Wang et al., 2011). Retirement adjustment is defined as “psychological comfort regarding the retirement life” (Wang et al., 2011, p. 204), which is often reflected in changes in the well-being of a retiree between the pre- and post-retirement stages (Gall et al., 1997). Wang et al. (2011) proposed the resource-based dynamic model to understand the quality of retirement adjustment over time. According to this model, retirement adjustment is a function of total resources during the transition. The amount of total resources influences the capabilities of retirees to meet the challenges during transition, which affect their physical and psychological well-being after retirement. There are three possible patterns of well-being outcomes: (i) Retirees will maintain their well-being if their total resources do not change significantly after retirement; (ii) Retirees will experience negative changes in well-being if their resources decline (e.g., loss of financial resources or connection with former colleagues); and (iii) Retirees will show an improvement in well-being if they acquire additional resources after retirement (e.g., making new friends or developing a new hobby). Therefore, every retiree features a unique pattern of retirement adjustment, depending on how the amount of total resources changes during transition.

In light of the theoretical framework of the resource-based dynamic model (Wang et al., 2011), Leung and Earl (2012) developed an assessment tool called the Retirement Resources Inventory (RRI) to measure various domains of individual resources. These resources include physical, financial, social, emotional, cognitive, and motivational resources discussed in the paper of Wang et al. (2011). The results of Leung and Earl’s (2012) factor analysis demonstrate that the six domains of resources can be categorized into three major groups: (1) Tangible resources include physical (e.g., perceived health and physical strength and illness) and financial resources (e.g., savings, investment, and perceived income adequacy); (2) Mental resources refer to resources that contribute to one’s mental capacity, including emotional (e.g., positive affect and emotional stability), cognitive (e.g., perceived control and memory capabilities), and motivational resources (e.g., perceived adaptability and flexibility in goal pursuit); and (3) Social resources pertain to types of social support and quality of social interaction.

Past studies focus mainly on a narrow set of retirement resources, particularly the physical and financial resources of retirees and their relationship with their spouse (e.g., Gall et al., 1997; Kim and Moen, 2002; Wang, 2007; van Solinge and Henkens, 2008). Other types of resources, such as mental resources, and other forms of social resources apart from spousal support, are given limited attention. Evidence indicate the beneficial effects of mental and social resources on post-retirement well-being. For example, perceived control and goal clarity (as mental resources) are positively correlated with post-retirement well-being and adjustment (Noone et al., 2009; Muratore and Earl, 2015). In addition to spousal support, support from family members and friends also predicts the well-being of retirees (Chou and Chi, 2003). To obtain a comprehensive assessment of the retirement resources possessed by the retirees, this study uses Leung and Earl’s (2012) RRI to measure the resource availability of retirees in various domains and examine the changes before and after retirement.

The amount of resources is expected to decrease after retirement. As retirees no longer have regular income after retirement (Atchley and Robinson, 1982), their financial security is reduced. They also lose social identity and self-worth derived from work and reduce their frequency of contact with former coworkers (Lo and Brown, 1999; Wong and Earl, 2009). Moreover, retirees face a number of challenges, such as age-related changes in physical health (Wang, 2007) and ways to spend spare time meaningfully and adapt to a new family role (Nuttman-Shwartz, 2004). Accordingly, this study hypothesizes that the total amount of retirement resources, including tangible, mental, and social resources, declines after retirement (H1).

To empirically test the propositions of the resource-based dynamic model (Wang et al., 2011), the present study investigates the relationships between changes in retirement resources and post-retirement well-being during transition. According to Wang et al.’s (2011) definition, successful adjustment occurs when a retiree experiences psychological comfort during transition. Indicators of psychological comfort include subjective well-being, as well as the physical, social, and mental aspects of well-being, and mental health. In the present research, post-retirement well-being is indicated by the levels of physical well-being, life satisfaction, and psychological well-being, as well as absence of psychological distress. It is hypothesized that changes in total retirement resources are positively associated with changes in post-retirement well-being (H2). Specifically, a decline in post-retirement well-being is observed among retirees with decreases in retirement resources, whereas an increase in post-retirement well-being is found among retirees with gains in resources.

### Pre-retirement Planning Activities

Pre-retirement planning is a goal-oriented behavior in which individuals devote effort to prepare for their retirement life. This behavior enables retirees to develop a realistic expectation of the changes to be experienced during transition (Peeters et al., 2008) and to set up a clear long-term goal for post-retirement life (Topa et al., 2009). Pre-retirement planning is often measured by a general term, such as self-perceived preparedness for retirement (Spiegel and Shultz, 2003). A few studies measuring specific domains of planning behaviors focus mainly on financial planning (e.g., Petkoska and Earl, 2009; Muratore and Earl, 2015). In addition to financial aspects, Law et al. (2006) identified four domains of retirement preparatory activities, namely, financial, health, social life, and psychological planning. Financial planning aids individuals to achieve financial security in later life, such as regular savings or property ownership. Health planning focuses on the maintenance of physical health, such as regular body check-up and physical exercises. Social life planning helps individuals to maintain and establish a supportive social network and to develop new and enjoyable hobbies for their post-retirement life. Psychological planning aims to promote psychological preparation for adjusting to potential changes after retirement, for example, attending pre-retirement preparation workshop or reading books on physical and psychological changes during retirement transition.

Empirical studies demonstrate that individuals who make retirement plans exhibit better retirement adjustment and post-retirement well-being. Specifically, the findings of both cross-sectional and longitudinal studies indicate that more pre-retirement planning activities are associated with better physical and psychological health (Wang, 2007; Yeung, 2013), positive attitudes and adjustment to retirement (Reitzes and Mutran, 2004; Muratore and Earl, 2015), and higher life satisfaction (Topa et al., 2009; Noone et al., 2013). However, the underlying mechanism between pre-retirement planning and post-retirement well-being remains largely unclear. Different types of preparatory activities may increase the levels of resources in the tangible, mental, and social domains; consequently, the quality of the retirees’ well-being over time may be improved (Wang and Shultz, 2010). For example, financial planning activities, such as savings or investment, contribute to the maintenance of financial resources (van Rooij et al., 2012). Health planning activities, such as regular exercises or physical check-up, may improve physical resources. Social life planning, such as development of a supportive social network, helps to increase social resources in post-retirement life.

Preparation for one domain may potentially encompass other domains of planning activities (Noone et al., 2009). For example, spousal discussion on retirement (as a form of psychological planning) facilitates the thoughts and planning of couples for financial and social domains, which consequently affects the financial, social, emotional, and motivational resources of retirees. Quick and Moen (1998) found that the individuals who performed more planning activities for retirement (regardless of the domain of planning) reported greater satisfaction with their retirement than those who planned less, because planning helps the retirees to develop realistic expectations about retirement (Pinquart and Schindler, 2007; Topa et al., 2009) and increase their preparedness in different domains (Hershey et al., 2003). These findings suggest that pre-retirement planning activities, regardless of domains of preparation, can help increase the amount of retirement resources in various domains. Therefore, this study also hypothesizes that more pre-retirement planning activities predict higher initial levels of retirement resources before retirement (H3a). This study also explores whether greater planning can help retirees to maintain their resources after retirement, and thus the rate of decline in resources may be smaller over time. Specifically, it is anticipated that more pre-retirement planning activities predict positive changes in resources over time (H3b).

The past findings reviewed above clearly demonstrate that pre-retirement planning positively contributes to retirement adjustment and post-retirement well-being. The present study takes a step forward in unveiling the underlying mechanism. By integrating the past literature on pre-retirement planning and the theoretical framework of the resource-based dynamic model, this study hypothesizes that the positive effect of pre-retirement planning on changes in post-retirement well-being is mediated by the amount of retirement resources possessed by retirees. Specifically, pre-retirement planning activities predict a higher initial level of retirement resources held by the retirees, which consequently affect the changes in post-retirement well-being over time (H4).

### Objectives and Design of the Study

Majority of past studies on retirement adjustment are largely conducted in the Western countries, such as the United States and Australia. These findings may not be fully applicable to Chinese retirees given the differences in retirement, pension, and social welfare systems between Hong Kong and other countries. For example, most of the working adults in Hong Kong are employed under a mandatory retirement scheme, which requires them to leave their job after a certain age, regardless of their job performance level. The typical retirement age in Hong Kong varies across sectors: 55 years for disciplinary forces (e.g., police officers or firefighters), 60 years for the public sector, and 65 years for private sectors. With a growing number of retirees in Hong Kong, their well-being after retirement should be investigated, and the factors influencing the level of post-retirement well-being should be identified. To the best of our knowledge, no local longitudinal study has systematically investigated the change trajectory in retirement resources and post-retirement well-being of Chinese retirees or explored the underlying mechanism between pre-retirement planning and post-retirement well-being. Therefore, this study intends to fill these knowledge gaps.

This study adopts the longitudinal design to measure intra-individual changes in resources and physical and psychological well-being of retirees during their transition to retirement. The transition to retirement refers to the period from being employed to completely leaving the job market. With reference to previous longitudinal studies on retirement (Richardson and Kilty, 1991; Reitzes and Mutran, 2004; Yeung, 2013), three waves of assessments were used in order to capture the change trajectory in retirement resources and post-retirement well-being over time. Time 1 assessment was conducted 6 months before the older workers retired from their full-time job, and Times 2 and 3 assessments were carried out 6 and 12 months, respectively, after actual retirement. The immediate assessment after actual retirement was excluded because it is usually the “honeymoon” period in which retirees enjoy their freedom of time and opportunities (Atchley, 1976). The intervals for Times 2 and 3 assessments enable us to test the initial and short-term effects of retirement resources on well-being.

With reference to prior literature (e.g., Shultz and Wang, 2011; Luhman et al., 2012; Yeung, 2013), post-retirement well-being is reflected in the levels of physical well-being, life satisfaction, and psychological well-being, as well as absence of psychological distress. Retirees experience poor adjustment to retirement when they show declines in physical functioning, life satisfaction, and psychological well-being and an increase in psychological distress over the three assessment points (Times 1–3).

**FIGURE 1:**
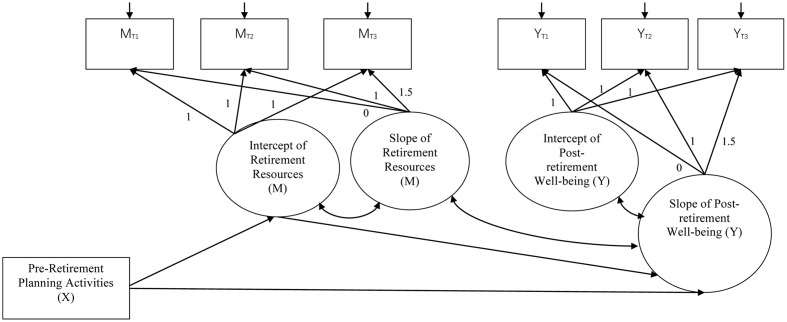
The proposed latent growth mediation model. X denotes the independent variable; M denotes the mediator; and Y denotes the dependent variable. The factor loadings of the intercept of retirement resources and post-retirement well-being were set to 1.0 on the three observed variables of the respective construct in Times 1–3. The factor loadings of the slope of retirement resources and post-retirement well-being were set to [0.0, 1.0, 1.5] on the three observed variables of the respective construct in Times 1–3 to reflect a linear change trajectory over a 1.5-year interval.

Latent growth model (LGM) was used to systematically examine changes in retirement resources and well-being outcomes over the three assessments (Liu et al., 2016). Inspired by Wang et al.’s (2011) resource-based dynamic model, the present study tests four hypotheses. First, the total amount of retirement resources, including tangible, mental, and social resources, are expected to decline after retirement. Second, changes in post-retirement well-being are anticipated to be positively associated with changes in retirement resources across the three assessments. Specifically, retirees with fewer resources after retirement experience a lower level of well-being. Third, total pre-retirement planning is expected to be associated with higher initial levels of retirement resources and positive changes in resources over time. Finally, the positive effect of pre-retirement planning on changes in post-retirement well-being is hypothesized to be mediated by the initial level of retirement resources. According to von Soest and Hagtvet (2011), this proposed mediation relationship via the intercept of resources is known as intercept-only mediation model. **Figure 1** illustrates the proposed latent growth mediation model for testing the fourth hypothesis.

## Materials and Methods

### Participants

This longitudinal study consisted of three phases of assessment: Time 1 was conducted 6 months before older workers retired from their full-time job, and Times 2 and 3 assessments were carried out 6 and 12 months, respectively, after the actual retirement of each participant. In the first assessment, 197 Chinese working adults (mean age = 58.7 years, *SD* = 3.59; 59.4% male) who were expected to retire in the next 6 months joined this research and completed the pre-retirement questionnaire. Among them, 136 participants were successfully contacted and completed the T2 post-retirement questionnaire (mean age = 59.4 years, *SD* = 3.36; 60.3% male). T3 contained 118 participants (mean age = 60.0 years, *SD* = 3.40; 61% male) who completed the follow-up questionnaire 12 months after retirement. The participation rate in T2 and T3 was 69% and 60% respectively. The participants who had completed the three assessments were similar to those who only joined the first assessment in terms of age [*t*(195) = 0.802], gender [χ^2^(197) = 0.323], education level [*t*(195) = 0.657], and occupation [χ^2^(197) = 0.123], *ns*. Most of the participants in the final sample (73.7%) worked as white-collar employees before retirement. About 62% of them completed secondary education, which is comparable with the average educational attainment of the local working population aged 50–65 years (Hong Kong Census and Statistics Department, 2011).

### Procedure

Human ethics approval was obtained from the research ethics committee of the affiliated university. Full-time employees who were expected to retire in the next 6 months were the target participants of this study. Employees who opted for early retirement scheme were excluded because their retirement decision could be affected by health or family issues, which subsequently influence their physical and psychological well-being after retirement.

The target participants were recruited through the human resources department of public and private organizations and through advertisements in a local newspaper. Invitation letters were sent to the head of the human resources department of corporations and companies in Hong Kong. Eleven organizations agreed to participate in this study. Upon their approval, an invitation letter, together with a questionnaire package, was distributed to the target employees. In addition, an advertisement was also posted on a local newspaper, which was freely distributed in public transportation. Eligible participants registered online or by phone to provide their contact information and their expected retirement date with their organization details. The questionnaire package was mailed to these participants after verifying their work status and retirement schedule. The participants who were recruited from different sources did not vary in age [*t*(195) = 0.777, *p* = 0.438], education level [*t*(195) = 1.110, *p* = 0.269], job position [*t*(195) = 0.301, *p* = 0.763], and major constructs measured in the present study. However, more female participants were recruited through the advertisement than those from the companies [χ^2^(197) = 10.956, *p* = 0.001].

A written informed consent was sought from each participant in the first assessment (T1). The participants were informed about the longitudinal nature of this study and were requested to provide contact information and the expected retirement date if they were interested in joining the follow-up assessments. The participants were contacted again 6 months (T2) and 12 months (T3) after their actual retirement. In all the three assessments, the participants completed the questionnaire and returned it to the researchers directly by post. Participants received a total sum of HKD400 (approximately USD 52) worth of supermarket vouchers as a token of appreciation for their participation in this longitudinal study.

### Measures

All the following measures were included in the three assessments, except pre-retirement planning activities that were measured only in T1. Except those already in Chinese, the measurement scales were translated into Chinese by two bilingual translators through the back-translation procedure. The Cronbach’s alphas of the measures of the three assessments are reported in **Table 1**.

**Table 1:** Descriptive statistics of Pre-retirement planning, retirement resources and Post-retirement well-being in Times 1–3.

	Time 1 (Pre-retirement)		Time 2 (6-month post-retirement)		Time 3 (12-month post-retirement)		Mean of the intercept (*SE*)	Mean of the slope (*SE*)	Variance of the intercept (*SE*)	Variance of the slope (*SE*)
	*M (SD)*	*α*	*M (SD)*	*α*	*M (SD)*	*α*				
**Pre-retirement Planning^a^ (0–20)**	9.06 (3.84)	0.74	–	–	–	–	–	–	–	–
**Retirement Resources**										
Total retirement resources (1–5)	3.36 (0.42)	0.92	3.31 (0.45)	0.93	3.33 (0.45)	0.89	3.36^∗∗∗^ (0.04)	-0.03^†^ (0.02)	0.18^∗∗∗^ (0.03)	0.02 (0.02)
Tangible resources (1–5)	3.47 (0.47)	0.79	3.42 (0.53)	0.81	3.41 (0.54)	0.85	3.47^∗∗∗^ (0.04)	-0.05^†^ (0.02)	0.20^∗∗∗^ (0.04)	0.04 (0.03)
Mental resources (1–5)	3.46 (0.47)	0.89	3.41 (0.48)	0.90	3.43 (0.49)	0.91	3.46^∗∗∗^ (0.04)	-0.02 (0.02)	0.20^∗∗∗^ (0.04)	0.03 (0.02)
Social resources (1–5)	2.80 (0.65)	0.85	2.76 (0.59)	0.81	2.77 (0.70)	0.81	2.81^∗∗∗^ (0.06)	-0.04 (0.04)	0.33^∗∗∗^ (0.47)	0.04 (0.50)
**Post-retirement well-being**										
Physical well-being (1–3)	2.73 (0.32)	0.87	2.68 (0.35)	0.88	2.71 (0.30)	0.86	2.73^∗∗∗^ (0.03)	-0.02 (0.02)	0.11^∗∗∗^ (0.02)	0.03^∗∗^ (0.01)
Life satisfaction (1–7)	5.01 (1.10)	0.88	4.83 (1.20)	0.91	4.96 (1.15)	0.91	4.98^∗∗∗^ (0.10)	-0.02 (0.06)	0.80^∗∗∗^ (0.24)	0.28^†^ (0.15)
Psychological well-being (1–5)	3.64 (0.47)	0.91	3.62 (0.48)	0.91	3.62 (0.46)	0.91	3.34^∗∗∗^ (0.04)	-0.01 (0.02)	0.32^∗∗∗^ (0.04)	0.03 (0.02)
Psychological distress (1–4)	1.76 (0.45)	0.89	1.79 (0.49)	0.89	1.82 (0.47)	0.87	1.76^∗∗∗^ (0.04)	0.04 (0.03)	0.19^∗∗∗^ (0.05)	0.07^∗∗^ (0.03)

*N = 118. The range of scores for each variable is shown in the parentheses on the first column. ^a^Pre-retirement planning activites were measured only in Time 1. ^∗^*p* < 0.05; ^∗∗^*p* < 0.01; ^∗∗∗^*p* < 0.001; ^†^*p* ≤ 0.06*.

#### Pre-retirement Planning Activities

In T1, participants were asked to report the types of planning activities that they performed for their retirement life. A locally developed measure of pre-retirement planning activities (Law et al., 2006; Yeung, 2013) was used, which covers preparatory behaviors in four domains, including financial (five items), health (four items), social life (four items), and psychological (seven items) planning. Following the rating format of Law et al.’s (2006) measure, a dichotomous rating scale was used (1 = *yes*; 0 = *no*), in which the participants were asked to indicate whether they performed any of the 20 retirement planning activities. Higher scores represent greater preparation for retirement. The dichotomous rating scale clearly reflects whether the participants had undertaken any of these activities shortly before their actual retirement, instead of their intention to perform these activities or not. A confirmatory factor analysis (CFA) was performed using MPlus 7 (Muthén and Muthén, 2012). Results of the CFA indicate that the goodness-of-fit of the one-factor model [AIC = 2580.599; BIC = 2762.738; χ^2^(170) = 195.570; CFI = 0.888; RMSEA = 0.042] was better than that of the four-factor model containing financial, health, social life, and psychological planning [AIC = 2628.810; BIC = 2808.231; χ^2^(164) = 245.782; CFI = 0.720; RMSEA = 0.067], Δχ^2^(6) = 50.212, *p* < 0.001. Therefore, in the present study, the sum of pre-retirement planning activities was computed and used in the following analyses in this study.

#### Retirement Resources

The RRI (Leung and Earl, 2012) consists of 35 items to measure three types of resources, namely, tangible resources (8 items), mental resources (18 items), and social resources (9 items). A sample item of tangible resources is “financial support from own savings.” Examples of mental resources are “experience positive emotions” and “have little control over the things that happen to me.” A sample item of social resource is “supportive interaction with friends.” Participants rated these items on a 5-point scale (1 = *very little* to 5 = *plenty*). Higher scores represent more resources possessed by the retirees. Results of the CFA showed that the goodness-of-fit of the three-factor model using Time 1 data [AIC = 8289.903; BIC = 8604.791; χ^2^(515) = 819.722; CFI = 0.828, RMSEA = 0.071] was better than that of the six-factor model which was originally proposed by Wang et al. (2011) [AIC = 8912.956; BIC = 9252.804; χ^2^(579) = 1014.160; CFI = 0.770, RMSEA = 0.080], Δχ^2^(64) = 194.438, *p* < 0.001; and the one-factor model [AIC = 8533.079; BIC = 8839.680; χ^2^(518) = 1068.897; CFI = 0.688, RMSEA = 0.095], Δχ^2^(3) = 249.175, *p* < 0.001. Therefore, the mean scores of tangible, social, and mental resources were computed. The resource-based dynamic model stresses that the level of post-retirement well-being is predicted by the total amount of resources held by the retirees (Wang et al., 2011). Therefore, the total resources were also calculated by averaging the mean scores of the three types of resources, with the assumption that these resources are equally important to each retiree.

#### Physical Well-being

Physical well-being was measured by the validated Chinese version of the Physical Functioning subscale of the Short-Form Health Survey (SF-36) (Ware and Sherbourne, 1992; Lam et al., 1998). Permission to use the SF-36 scale was obtained. The participants rated the 10 items on a 3-point scale (1 = *limited a lot* to 3 = *not limited at all)* to reflect whether their daily activities were limited by their health. Higher scores represent better physical well-being.

#### Life Satisfaction

The Chinese version of the Life Satisfaction Scale was utilized (Diener et al., 1985; Sachs, 2003). This scale consists of five items to assess the retiree’s general satisfaction with life. The participants rated each item using a 7-point Likert scale (1 = *strongly disagree* to 7 = *strongly agree*). Higher scores denote higher satisfaction with life.

#### Psychological Well-being

The validated Chinese version of Ryff’s (1989) psychological well-being was employed in the present study (Cheng and Chan, 2005). A sample item of this scale is “Some people wander aimlessly through life, but I am not one of them.” The participants rated the 24 items on a 5-point Likert scale (1 = *strongly disagree* to 5 = *strongly agree*). Higher scores indicate better psychological health.

#### Psychological Distress

The validated Chinese version of the 12-item General Health Questionnaire (Shek, 1989) was adopted to assess the participants’ psychological distress such as depression, social dysfunction, and loss of confidence in the past 4 weeks. The participants rated the items on a 4-point scale, with higher scores representing more severe psychological distress.

#### Demographic Variables and Covariates

Age, gender, and education level were recorded in T1. The occupation prior to retirement was also recorded, with 1 = *white-collar workers* and 0 = *service-oriented workers or technicians*. Preliminary analyses showed that the education level and occupation before retirement did not significantly correlate with the four well-being variables. Therefore, these two variables were excluded from the following analyses.

### Analytical Plan

This study involves changes in retirement resources and post-retirement well-being across the three assessments, therefore, the LGM is employed to examine the changes in the levels of these variables and their relationships over time. The critical information obtained from the LGM is the parameter estimations of two latent factors for each construct: the *mean values* of the intercept and slope factors (which represent the magnitude of the variable’s initial level and rate of change over time, respectively) and the *variances* in the intercept and slope factors (which represent the individual variations in the variable’s initial level and rate of change, respectively) (Liu et al., 2016). H1 is examined by assessing whether the mean value of the latent slope of retirement resources would be significant. H2 is tested by assessing the slope–slope correlations between the retirement resources and post-retirement well-being. The LGM is also adopted to test H3 regarding the effect of pre-retirement planning on the initial level (i.e., intercept) and rate of change (i.e., slope) in retirement resources over time.

H4, which pertains to the proposed mediating effect of retirement resources on the relationship between pre-retirement planning and post-retirement well-being, is examined by the latent growth mediation model suggested by Selig and Preacher (2009). In particular, the effect of the pre-retirement planning activities on the change in post-retirement well-being through the initial level of retirement resources is investigated.

## Results

### Descriptive Analyses

**Table 1** presents the mean, standard deviations, and Cronbach’s alphas of each construct measured in T1–3. **Table 2** shows the correlation coefficients among pre-retirement planning, retirement resources, and post-retirement well-being. Pre-retirement planning was significantly correlated with greater total retirement resources in T1–3 (*r* = 0.21–0.24, *p* < 0.05) and several of the post-retirement well-being variables (e.g., psychological well-being and psychological distress in T2 and life satisfaction in T2 and T3). Tangible, mental, and social resources, as well as the total resources in T1, were significantly associated with the four well-being variables in T2 and T3.

**Table 2:** Correlation coefficients among major variables.

	1	2	3	4	5	6	7	8	9	10	11	12	13	14	15	16	17	18	19	20	21	22	23	24
(1) Planning	–																							
(2) T1 TRR	**0.21**	–																						
(3) T1 Tangible	0.05	**0.76**	–																					
(4) T1 mental	**0.19**	**0.91**	**0.53**	–																				
(5) T1 social	**0.30**	**0.69**	**0.32**	**0.51**	–																			
(6) T1 PHY	0.01	**0.31**	**0.38**	**0.32**	0.05	–																		
(7) T1 LS	0.11	**0.57**	**0.50**	**0.47**	**0.36**	0.17	–																	
(8) T1 PWB	**0.25**	**0.76**	**0.52**	**0.71**	**0.54**	**0.24**	**0.64**	–																
(9) T1 GHQ	-0.04	**-0.66**	**-0.56**	**-0.59**	**-0.39**	**-0.24**	**-0.48**	**-0.62**	–															
(10) T2 TRR	**0.24**	**0.86**	**0.70**	**0.76**	**0.58**	**0.31**	**0.53**	**0.68**	**-0.57**	–														
(11) T2 Tangible	0.17	**0.70**	**0.76**	**0.54**	**0.38**	**0.28**	**0.50**	**0.52**	**-0.45**	**0.85**	–													
(12) T2 Mental	0.17	**0.83**	**0.59**	**0.81**	**0.50**	**0.35**	**0.45**	**0.65**	**-0.59**	**0.92**	**0.64**	–												
(13) T2 Social	**0.36**	**0.63**	**0.41**	**0.48**	**0.71**	0.10	**0.41**	**0.52**	**-0.38**	**0.77**	**0.54**	**0.60**	–											
(14) T2 PHY	-0.00	**0.37**	**0.43**	**0.30**	0.08	**0.70**	**0.35**	**0.35**	**-0.21**	**0.46**	**0.49**	**0.43**	**0.22**	–										
(15) T2 LS	**0.21**	**0.52**	**0.41**	**0.43**	**0.39**	**0.20**	**0.58**	**0.52**	**-0.42**	**0.62**	**0.57**	**0.53**	**0.51**	**0.32**	–									
(16) T2 PWB	**0.22**	**0.76**	**0.56**	**0.89**	**0.56**	**0.24**	**0.52**	**0.81**	**-0.59**	**0.76**	**0.56**	**0.77**	**0.59**	**0.36**	**0.68**	–								
(17) T2 GHQ	**-0.19**	**-0.56**	**-0.47**	**-0.49**	**-0.34**	**-0.20**	**-0.43**	**-0.56**	**0.65**	**-0.64**	**-0.53**	**-0.65**	**-0.40**	**-0.39**	**-0.50**	**-0.66**	–							
(18) T3 TRR	**0.21**	**0.80**	**0.64**	**0.71**	**0.55**	**0.28**	**0.52**	**0.66**	**-0.56**	**0.85**	**0.76**	**0.77**	**0.60**	**0.47**	**0.62**	**0.76**	**-0.64**	–						
(19) T3 tangible	0.14	**0.67**	**0.71**	**0.51**	**0.42**	**0.27**	**0.46**	**0.46**	**-0.46**	**0.73**	**0.82**	**0.59**	**0.46**	**0.40**	**0.55**	**0.56**	**-0.53**	**0.86**	–					
(20) T3 mental	0.16	**0.73**	**0.51**	**0.74**	**0.40**	**0.31**	**0.47**	**0.66**	**-0.56**	**0.77**	**0.62**	**0.81**	**0.45**	**0.51**	**0.54**	**0.72**	**-0.64**	**0.90**	**0.67**	–				
(21) T3 social	**0.23**	**0.52**	**0.31**	**0.39**	**0.62**	0.02	**0.31**	**0.40**	**-0.26**	**0.52**	**0.43**	**0.38**	**0.63**	0.13	**0.38**	**0.51**	**-0.31**	**0.64**	**0.42**	**0.37**	–			
(22) T3 PHY	-0.02	**0.36**	**0.41**	**0.31**	0.10	**0.63**	**0.27**	**0.38**	**-0.32**	**0.43**	**0.48**	**0.39**	**0.21**	**0.75**	**0.34**	**0.42**	**-0.45**	**0.51**	**0.78**	**0.53**	0.16	–		
(23) T3 LS	**0.25**	**0.60**	**0.51**	**0.50**	**0.42**	**0.24**	**0.58**	**0.63**	**-0.48**	**0.66**	**0.63**	**0.57**	**0.51**	**0.44**	**0.80**	**0.72**	**-0.58**	**0.75**	**0.68**	**0.67**	**0.43**	**0.52**	–	
(24) T3 PWB	0.15	**0.66**	**0.49**	**0.62**	**0.43**	**0.25**	**0.55**	**0.77**	**-0.53**	**0.70**	**0.59**	**0.66**	**0.52**	**0.43**	**0.63**	**0.82**	**-0.65**	**0.76**	**0.58**	**0.76**	**0.42**	**0.52**	**0.75**	–
(25) T3 GHQ	-0.07	**-0.53**	**-0.52**	**-0.45**	**-0.26**	**-0.32**	**-0.38**	**-0.49**	**0.54**	**-0.59**	**-0.55**	**-0.55**	**-0.34**	**-0.50**	**-0.38**	**-0.52**	**0.73**	**-0.64**	**-0.56**	**-0.65**	**-0.24**	**-0.59**	**-0.54**	**-0.59**

*N = 118. T1 = Time 1; T2 = Time 2; T3 = Time 3; Planning = Pre-retirement planning activities; TRR = Total retirement resources; Tangible = Tangible resources; Mental = Mental resources; Social = Social resources; PHY = Physical well-being; LS = Life satisfaction; PWB = Psychological well-being; GHQ = Psychological distress. Correlation displayed in bold are significant at *p* < 0.05*.

### Changes in Retirement Resources and Post-retirement Well-being

The LGM was conducted on each of the retirement resources and post-retirement well-being without inclusion of any covariate. Following the steps of Liu et al. (2016), in the following LGM analyses, the factor loadings of the intercept of each construct were set at 1.0 on the three observed variables in T1–3 (e.g., composite scores of psychological distress measured in the three time points). The factor loadings of the slope were set at [0.0, 1.0, 1.5]^1^ on the three observed variables to reflect a linear change trajectory over a 1.5-year interval. All of these models have satisfactory fit, wherein the CFI ranged from 0.977 to 1.00 and the RMSEA from 0.000 to 0.087.

The last four columns in **Table 1** present the mean values and variances of the intercept and slope factors of retirement resources and post-retirement well-being. LGM was performed to test H1 on whether retirement resources decline over time. Results demonstrate that tangible resources show a marginally significant mean value of the slope factor (slope mean = -0.04, *p* = 0.059; T1–3 means = 3.47, 3.42, and 3.41, respectively), which suggests that the mean level of tangible resources show a decreasing trend over time. The slope of total resources was also marginally significant (slope mean = -0.03, *p* = 0.060; T1–3 means = 3.36, 3.31, and 3.33, respectively), suggesting a trend of fewer overall resources after retirement. The means and variances of the slope of mental and social resources were not statistically significant, which suggest that these two resources remained largely stable during transition. However, the variances in the intercept factor of all the four resource variables were significantly, which indicate that individual differences exist in the retirement resources at T1. Therefore, H1 is partially supported.

The mean values of the slope of the four post-retirement well-being variables were not significant. However, the negative values for the slope of physical well-being, psychological well-being, and life satisfaction, as well as the positive value for the slope of psychological distress imply that there is a general trend of poorer well-being during the retirement transition. The variances in the slope of physical well-being (slope variance = 0.03, *p* = 0.004; T1–3 means = 2.73, 2.68, and 2.71, respectively) and psychological distress (slope variance = 0.07, *p* = 0.005; T1–3 means = 1.76, 1.79, and 1.82, respectively) were significant, whereas that of life satisfaction (slope variance = 0.28, *p* = 0.057; T1–3 means = 5.01, 4.83, and 4.96, respectively) was marginally significant, indicating that there were individual differences in the rate of change in these three well-being variables.

### Relationship between Retirement Resources and Post-retirement Well-being

Two sets of LGM were conducted separately on each of the four well-being variables to test H2 whether changes in retirement resources are correlated with changes in post-retirement well-being: (1) The slope of total resources was correlated with the slope of the well-being variable; and (2) The slopes of tangible, social, and mental resources were correlated with the slope of the well-being variable. In these analyses, the initial levels of the resources and well-being variables were also included.

In the LGM with total retirement resources, the slope of total resources was positively correlated with the slopes of physical well-being (*r* = 0.01), life satisfaction (*r* = 0.04), and psychological well-being (*r* = 0.02), and negatively correlated with that of psychological distress (*r* = -0.02), *p*s ≤ 0.001. These results suggest that the changes in total retirement resources are positively associated with the change trajectory in post-retirement well-being over time.

In the LGM with all the three types of retirement resources, the slopes of tangible, mental, and social resources were significantly correlated with the slope of psychological well-being (*r* = 0.01, *p* = 0.005; *r* = 0.02, *p* < 0.001, and *r* = 0.02, *p* = 0.008, respectively). The slopes of tangible and mental resources were significantly correlated with the slopes of physical well-being (*r* = 0.01, *p* = 0.002; and *r* = 0.02, *p* < 0.001, respectively), life satisfaction (*r* = 0.05, *p* = 0.001; and *r* = 0.04, *p* = 0.004, respectively), and psychological distress (*r* = -0.02, *p* = 0.015; and *r* = -0.02, *p* < 0.001, respectively). The slope of social resources did not correlate with the slopes of physical well-being (*r* = -0.002, *p* = 0.687), life satisfaction (*r* = 0.02, *p* = 0.432), and psychological distress (*r* = -0.02, *p* = 0.101).

These findings reveal that retirees with increased resources after retirement, especially in the domains of tangible and mental resources, are more likely to experience better physical and psychological well-being, higher life satisfaction, and a lower level of psychological distress during their transition to retirement. Therefore, H2 is largely supported.

### The Effect of Pre-retirement Planning on Retirement Resources

H3 concerns the effect of pre-retirement planning on the initial level and slope of retirement resources. Results of the LGM reveal that total pre-retirement planning significantly predicted of the initial levels of the total resources (*B* = 0.06, *SE* = 0.02, *p* = 0.019), and social and mental resources (*B* = 0.05, *SE* = 0.02, *p* < 0.001; and *B* = 0.02, *SE* = 0.01, *p* = 0.040, respectively), but not tangible resources (*B* = 0.01, *SE* = 0.01, *p* = 0.509). Therefore, H3a is largely supported. However, the effect of pre-retirement planning on the slopes of total resources (*B* = 0.02, *SE* = 0.03) and the three resources types (tangible: *B* = 0.01, *SE* = 0.01; mental: *B* = -0.00, *SE* = 0.01, and social: *B* = -0.00, *SE* = 0.01) was not significant. Therefore, H3b is not supported.

### Mediating Role of Retirement Resources

H4 tests whether the effect of pre-retirement planning activities on the changes in post-retirement well-being is mediated by the initial level of retirement resources. The intercept-only mediation model was executed following the LGM framework of Selig and Preacher (2009) and von Soest and Hagtvet (2011). The effect of *X* (pre-retirement planning) on the slope of *Y* (post-retirement well-being) is mediated by the intercept of *M* (retirement resources) (**Figure 1**). In the mediation model, the paths from X to the intercept of M, from the intercept of M to the slope of Y, and from X to the slope of Y were tested. The covariance between the slopes of M and Y were also included as the the resource-based dynamic model expects the changes in well-being are correlated with the change in resources. Two sets of the latent growth mediation models were performed separately on each post-retirement well-being variable: one contains the total retirement resources as the mediator (*M*), whereas the other contains the three types of retirement resources as the mediators. Age and gender were controlled as covariates in the model.

**FIGURE 2:**
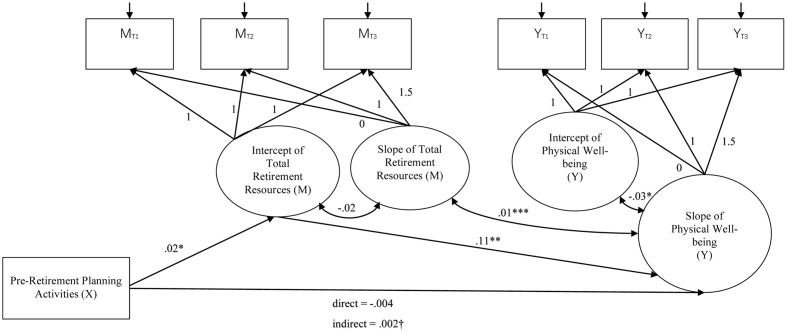
The latent growth mediation model on physical well-being. X denotes the independent variable; M denotes the mediator; and Y denotes the dependent variable. The model fit indices are: CFI = 0.972; RMSEA = 0.083; AIC = 98.075; BIC = 167.342. Age and gender were controlled in the model as covariates. ^∗^*p* < 0.05; ^∗∗^*p* < 0.01; ^∗∗∗^*p* < 0.001; †*p* = 0.063.

**FIGURE 3:**
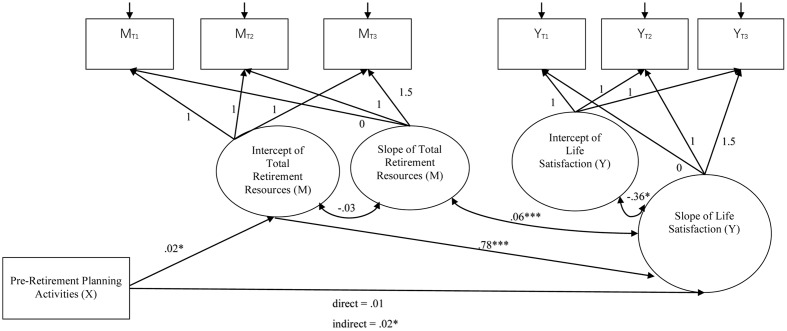
The latent growth mediation model on life satisfaction. X denotes the independent variable; M denotes the mediator; and Y denotes the dependent variable. The model fit indices are: CFI = 0.921; RMSEA = 0.146; AIC = 998.979; BIC = 1072.017. Age and gender were controlled in the model as covariates. ^∗^*p* < 0.05; ^∗∗^*p* < 0.01; ^∗∗∗^*p* < 0.001.

**FIGURE 4:**
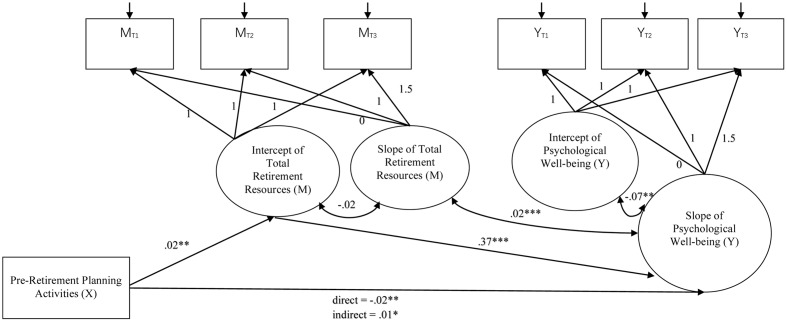
The latent growth mediation model on psychological well-being. X denotes the independent variable; M denotes the mediator; and Y denotes the dependent variable. The model fit indices are: CFI = 0.901; RMSEA = 0.150; AIC = 250.945; BIC = 325.754. Age and gender were controlled in the model as covariates. ^∗^*p* < 0.05; ^∗∗^*p* < 0.01; ^∗∗∗^*p* < 0.001.

**FIGURE 5:**
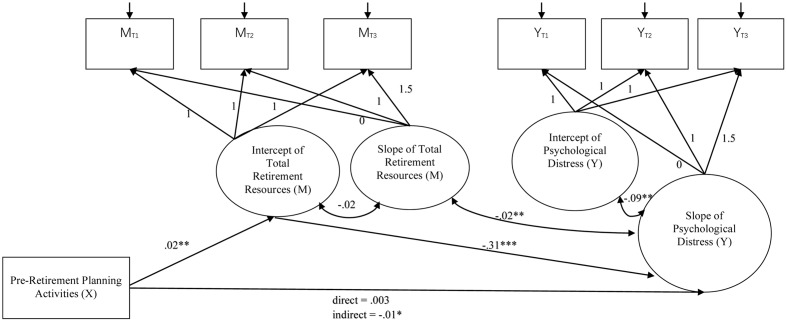
The latent growth mediation model on psychological distress. X denotes the independent variable; M denotes the mediator; and Y denotes the dependent variable. The model fit indices are: CFI = 0.909; RMSEA = 0.154; AIC = 389.006; BIC = 460.823. Age and gender were controlled in the model as covariates. ^∗^*p* < 0.05; ^∗∗^*p* < 0.01; ^∗∗∗^ *p* < 0.001.

The latent growth mediation model with the intercept of total retirement resources as the mediator^2^ showed a significant indirect effect of pre-retirement planning on the slopes of life satisfaction (*B* = 0.02, *SE* = 0.02, *p* = 0.030), psychological well-being (*B* = 0.01, *SE* = 0.004, *p* = 0.021), and psychological distress (*B* = -0.01, *SE* = 0.003, *p* = 0.024). The indirect effect on the physical well-being was marginally significant (*B* = 0.002, *SE* = 0.001, *p* = 0.063). **Figures 2–5** show the unstandardized coefficients of the latent growth mediation model on the four well-being variables.

Results of the latent growth mediation model with the intercept of the three retirement resources as the mediators reveal that the total indirect effect of pre-retirement planning on the slopes of psychological well-being (*B* = 0.01, *SE* = 0.004, *p* = 0.032) and life satisfaction (*B* = 0.02, *SE* = 0.01, *p* = 0.042) was significant. In particular, the initial level of social resources mediated the positive effect of pre-retirement planning on the increase in psychological well-being (*B* = 0.01, *SE* = 0.003, *p* = 0.010) and life satisfaction (*B* = 0.03, *SE* = 0.01, *p* = 0.005). The indirect effect through tangible and mental resources was not significant. The latent growth mediation model on physical well-being (*B* = 0.003, *SE* = 0.003, *p* = 0.263) and psychological distress (*B* = -0.002, *SE* = 0.01, *p* = 0.662) through the three types of retirement resources was not significant.

Combining the two sets of mediation analyses together, the results suggest that pre-retirement planning activities are associated with a larger total amount of resources (particularly social resources) possessed by the retirees in T1, which then contribute to positive changes in their psychological well-being and life satisfaction after retirement. Therefore, H4 is partially supported.

## Discussion

This three-wave longitudinal study systematically examined changes in the retirement resources and psychological and physical well-being of Hong Kong Chinese retirees before and after their actual retirement. Consistent with the proposition of the resource-based dynamic model (Wang et al., 2011), the results of the LGMs show that post-retirement well-being depends on the changes in the total resources during the transition. The findings of the latent growth mediation models also reveal that the beneficial effect of pre-retirement planning activities on the changes in post-retirement well-being can be explained by the initial amount of total resources possessed by the retirees.

### Changes in Retirement Resources and Post-retirement Well-being and Their Association

Retirement is often perceived as a stressful life event that causes a wide range of negative psychosocial consequences, such as psychological distress, loneliness, and physical health problems (e.g., Dave et al., 2006; Wang, 2007; Wong and Earl, 2009). However, some researchers argue that retirement is not necessarily traumatic and stressful (Kim and Moen, 2002; Fehr, 2012), and not every retiree experiences a decrease in his/her physical and psychological well-being (Wang, 2007). This study provides a more in-depth examination of changes in post-retirement well-being by following the retirees in their transition period. The findings of the present study reveal that the retirees in this sample can maintain their psychological and physical well-being 1 year after their actual retirement. However, the variances in the slope of physical well-being and psychological distress are significant, whereas that of life satisfaction is marginally significant, implying that individual variations exist in the rate of changes in these well-being outcomes over time. For example, comparing the changes in physical well-being 6 and 12 months after retirement shows that over 43% of the retirees first experienced decreases 6 months after retirement (T2). Among these participants, only 16% of them continued to experience decreases in physical functioning but the majority could maintain and even improve their physical health 1 year after retirement (T3). These results support the proposition of the resource-based dynamic model that retirement adjustment takes multiple forms, which can be positive, negative, or neutral, and the pattern of changes varies over time.

The resource-based dynamic model (Wang et al., 2011) emphasizes the importance of the resources possessed by each retiree in determining his/her quality of adjustment and level of well-being after retiring from the full-time job. This study examines the changes in the total retirement resources, including tangible, mental, and social resources, in Times 1–3. A trend of fewer total resources, which mainly arise from the domain of tangible resources, is observed in the current sample of retirees. After retiring from the full-time employment, the retirees do not have regular income and have to rely on their private savings to support their living and medical expenses. Chou et al. (2015) commented that even the individuals with substantial financial resources continue to worry about their financial adequacy after retirement. The decrease in financial resources is particularly common in Hong Kong because no pension system exists for most retirees (except civil servants), and the government only provides a minimal amount of allowance to the aged population. For resources in the mental and social domains, such as cognitive capabilities, motivation or social support, no significant change is observed in the present sample, suggesting that the retirees can maintain the resources in these two domains after retiring from their jobs. Since this study only assesses the changes in resources 1 year after actual retirement, so the long-term changes remain largely unknown. Future studies should extend the present research to a longer interval (e.g., 5 years) to obtain a clear picture on the changes in retirement resources over time.

This research advances the current literature on retirement adjustment by empirically testing the propositions of the resource-based dynamic model (Wang et al., 2011). In particular, the association between changes in retirement resources and well-being in Times 1–3 was examined. Consistent with the model prediction, the retirees with increased total resources during the transition reported positive changes in their physical well-being, life satisfaction, and psychological well-being, as well as lower psychological distress across the three assessments. A reverse pattern of relationship is observed when the retirees reported a decrease in the total resources. Past studies that use the RRI demonstrated the effect of retirement resources on the retrospective recall of retirement adjustment and satisfaction in a sample of Australian retirees (Leung and Earl, 2012). The present study took a step further to investigate the longitudinal effect of resource changes on changes in physical and psychological well-being during the transition to retirement. Retirees, who reported gains in retirement resources, particularly tangible and mental resources, can better meet the challenges in the transition period, which consequently contribute to better well-being after retirement. These findings reveal the importance of overall resource availability in determining the well-being of retirees.

### The Mediating Effect of Retirement Resources

Pre-retirement planning activities are important to the successful adaptation to this critical life event. Past cross-sectional and longitudinal studies clearly demonstrate the positive effects of planning behaviors on post-retirement well-being, such as life satisfaction and psychological well-being (e.g., Wang, 2007; Donaldson et al., 2010; Yeung, 2013; Muratore and Earl, 2015). The current study investigated the underlying mechanism of such positive relationship between pre-retirement planning and post-retirement well-being. A mediation model was proposed: preparation for retirement is associated with higher initial levels of retirement resources, which consequently contribute to positive changes in well-being over time. The results of the latent growth mediation models support our prediction. In particular, overall planning activities for retirement contribute to a higher initial level of total resources possessed by the retirees in T1, which enable them to cope with challenges and negative experiences during the transition and consequently maintain and preserve their well-being 1 year after retirement. By examining the three types of retirement resources in the LGM, this study reveals that among various types of resources possessed by the retirees, the initial level of social resources largely accounts for the positive effect of pre-retirement planning on the changes in psychological well-being and life satisfaction. The retirees with more planning activities tend to have more social support from family members and friends, which then facilitate a positive change in their psychological well-being and life satisfaction. These findings help advance the current literature on pre-retirement planning by identifying the paths that lead to better post-retirement well-being.

Retirement planning facilitates a realistic expectation of retirement experiences and promotes goal setting for post-retirement life among retirees. Different types of preparatory behavior, such as psychological preparation for post-retirement changes, seeking for social support, savings and investment, and regular physical exercises, help the retirees to maintain or improve their resources availability. Past research focuses largely on financial and health planning activities, making other aspects of preparation being less emphasized. The findings on the mediating role of social resources in the present research suggest that future pre-retirement planning programs should put more effort to strengthen the amount of social resources of retirees, such as increase their social support network and social participation, to facilitate successful adjustment to retirement.

### Limitations and Future Directions

A few limitations should be considered when interpreting the findings reported in this paper. First, this study was conducted with a small sample of Hong Kong Chinese retirees, so the findings may not be generalized to retirees of other countries because of the differences in their retirement and social welfare systems. However, this study reveals the underlying mechanism of pre-retirement planning and post-retirement well-being, thus provides insights on the design of future pre-retirement planning programs. Future studies should examine retirement adjustment in a larger sample, preferably with occupational stratification, to systematically understand the impact of retirement on retired persons. Second, this 18-month longitudinal study could only demonstrate the short-term effects of pre-retirement and total resources on well-being. Their long-term predictive values await further investigation. Third, only the planning activities 6 months before retirement were recorded. Some retirees may possibly perform more preparatory behaviors (e.g., attending a pre-retirement workshop 1 month before their retirement) when the actual retirement date becomes closer, which then affects the accuracy of the amount of total resources before retirement. Future research may include a follow-up assessment around the actual retirement to accurately measure the pre-retirement planning of the retirees. Fourth, this study relied on the self-reported questionnaires. Future studies should include an objective assessment of the retirees’ resources and well-being, such as others’ rating of social support or an objective measure of physical health.

## Conclusion

This longitudinal study investigated the changes in retirement resources and post-retirement well-being of Hong Kong Chinese retirees for 18 months. The retirees in the present sample can maintain their physical and psychological well-being after retiring from their jobs, though significant individual variations in the change rate in physical well-being and psychological distress are observed. A trend of decreasing tangible resources after retirement is also noted. The changes in post-retirement well-being are closely related to the changes in the total retirement resources over time. The latent growth mediation analyses also reveal that the beneficial effects of pre-retirement planning activities on the changes in psychological well-being and life satisfaction can be explained by the retirees’ initial levels of total resources, particularly resources in the social domain. Future pre-retirement planning programs should therefore strengthen the amount of social resources possessed by the retirees.

## Ethics Statement

This study was carried out in accordance with the recommendations of Human Subjects Ethics Sub-Committee at City University of Hong Kong, with written informed consent from all participants. The protocol was approved by the Human Subjects Ethics Sub-Committee at City University of Hong Kong.

## Author Contributions

DY is responsible for designed and conducted the study, analyzed the data, and wrote the manuscript. XZ is responsible for conducted the literature review and wrote the manuscript.

## Conflict of Interest Statement

The authors declare that the research was conducted in the absence of any commercial or financial relationships that could be construed as a potential conflict of interest.

## Footnotes

1 In this study, fixed loadings [0, 1, 1.5] are used to represent the time scores of the latent slope factor (Muthén and Muthén, 2012). Preliminary analyses comparing the result pattern between the linear growth model and the freely-estimated growth model show that the models with fixed slopes are not significantly worse than the models with freely-estimated slope parameters. Therefore, this study reports the results of the fixed slope models.

2 Exploratory analyses were performed to test whether the slope of resources is also a significant mediator. In the slope-only mediation model, no significant indirect effect through the slope of total resources was found on the relationship between pre-retirement planning and post-retirement well-being (physical well-being: *B* = 0.001, *SE* = 0.002, *p* = 0.625; life satisfaction: *B* = 0.003, *SE* = 0.006, *p* = 0.620; psychological well-being: *B* = 0.002, *SE* = 0.003, *p* = 0.599; and psychological distress: *B* = -0.002, *SE* = 0.003, *p* = 0.589).
